# Spatial Association Effect of Haze Pollution in Cheng-Yu Urban Agglomeration

**DOI:** 10.1038/s41598-020-66665-8

**Published:** 2020-06-16

**Authors:** Degang Zhang, Yuanquan Lu, Yuan Tian

**Affiliations:** 1 School of Economics and Management, Chongqing Normal University, Chongqing, 401331 China; 2 School of Economics, Chongqing Technology and Business University, Chongqing, 400067 China

**Keywords:** Atmospheric dynamics, Climate change

## Abstract

This study takes a network perspective to examine the spatial spillover effects of haze pollution in Cheng-Yu urban agglomeration which is the fourth largest urban agglomeration and a comprehensive demonstration zone of new urbanization in China. Firstly, we use Granger causality test to construct haze pollution spatial association network, and then we apply social network analysis to reveal the structural characteristics of this network. The results show that: haze pollution in Cheng-Yu urban agglomeration is a complex multithreaded network. Chongqing, Chengdu, Guang’an, Luzhou, Deyang and Nanchong are the centers of the network, sending and transmitting the most relationships. The haze pollution spatial association network can be divided into net beneficiary block, net overflow block, bilateral overflow block and broker block. These four blocks present obvious geographical distribution characteristics and are partly related to the difference of urbanization. The above results contribute by illustrating the current spatial spillover situation of haze pollution and provide a theoretical foundation for the government that it should simultaneously consider cities’ statues and their spatial spillover effects in the network rather than simple geographic proximity when formulating future haze pollution control policies in urban agglomeration.

## Introduction

Rapid industrialization and urbanization have also produced serious air pollution when driving China’s economic growth. In recent years, the extreme haze pollution that frequently erupted in economically developed urban agglomerations has severely affected people’s production and life^1–4^. Haze pollution has become a top priority for the Chinese government’s environmental governance^5^.

Environmental science research based on air quality models has confirmed that haze pollution is highly spatially contagious. For example, Fan *et al*.^6^, and Qin *et al*.^7^, used the CMAQ model to simulate the air pollution in the Pearl River Delta region. Jiang *et al*.^8^, and Wang *et al*.^9^, used the GRAPES-CUACE model to simulate the haze pollution in the Jing-Jin-Ji area. In addition, there were many literatures that use air quality models to analyze regional transport of air pollution^10–12^. These studies have shown that regional air pollution can indeed be transmitted across the boundary under the influence of atmospheric circulation. However, due to the short time span of sample, these studies cannot examine the spillover effects of urban air pollution from a dynamic perspective. At the same time, it is impossible to reveal the network structure characteristics of air pollution and reduce the application value^13^.

In recent years, some studies have combined spatial statistical techniques with econometrics to reveal the spatial distribution and spillover effects of haze pollution. Zheng *et al*.^14^, analyzed the statistical characteristics of the spatial correlation of haze pollution in seven cities around Beijing, and used the Granger causality test to reveal the spatial spillover of haze pollution between cities. Hu *et al*.^15^, used the Moran’s I index and spatial econometric model to analyze the spatial agglomeration and spillover effects of PM_2.5_ in 367 cities in China. Yan *et al*.^16^, analyzed the temporal and spatial evolution characteristics of PM_2.5_ in Jing-Jin-Ji, and found that PM_2.5_ pollution in Jing-Jin-Ji area has significant spatial spillover effects. Ma *et al*.^17^, studied the spatial characteristics of haze pollution in the Yangtze River Economic Belt and found a positive spatial correlation and significant spillover effect of haze pollution. In addition, there were many literatures that use spatial econometric model to analyze the spillover effect of haze pollution^18,19^. These studies have revealed the spatial spillover effects of haze pollution to a certain extent, but neglected the complicated network structure characteristics of haze pollution in urban agglomeration. In fact, only by revealing the functions of different cities in the haze pollution spatial association network, it is possible for cities to develop effective joint prevention and control policies^20^. Therefore, this study aims to take a network perspective to examine the spatial spillover effects of haze pollution.

Different from most of the existing studies which focus on haze pollution in Jing-Jin-Ji, Yangtze River Delta and Pearl River Delta, this study takes Cheng-Yu urban agglomeration as the research object. Cheng-Yu urban agglomeration is the fourth largest urban agglomeration and a comprehensive demonstration zone for new urbanization in China. It covers an area of 185,000 square kilometers, and has a population of 95 million permanent residents. Its gross regional product (GRP) amounted 5.7 trillion yuan in 2018. The extreme haze pollution has been outbreak frequently in Cheng-Yu urban agglomeration since 2013. In the past year of 2018, the average concentration of PM_2.5_ in Cheng-Yu urban agglomeration is 42.46 ug/m^3^, higher than the average concentration of PM_2.5_ in China overall 39 ug/m^3^. The situation of preventing and controlling haze pollution is still very serious. However, the existing literatures have payed obviously insufficient attention to haze pollution in Cheng-Yu urban agglomeration. In order to control haze pollution effectively, it is necessary to clarify the spatial association characteristics of the haze pollution in Cheng-Yu urban agglomeration.

Compared with the existing literatures, the marginal contribution of this study is reflected in the following three aspects. Firstly, we take a network perspective to examine the spatial spillover effects in Cheng-Yu urban agglomeration. It breaks through the traditional spatial analysis of geographical proximity and helps to reveal the spatial spillover of haze pollution more comprehensively. Secondly, we use Granger causality test method to construct haze pollution spatial association network between two cities in Cheng-Yu urban agglomeration. Granger causality test has a strong econometric explanation that can disclose how haze pollution of cities affects each other, if the current haze pollution of y city can be explained by past haze pollution of x city, it implies that haze pollution of x city causes haze pollution of y city. Meanwhile, we use social network analysis to reveal the characteristics of haze pollution spatial association network, which helps to find out the network center city and the transmission media city in haze pollution spatial association network of Cheng-Yu urban agglomeration. These new methods application make the research conclusions more reliable and innovative. Thirdly, we provide a new perspective of haze pollution control policy that the key to the joint prevention and control of haze pollution in urban agglomeration lies in the control of network central city and key transmission node city, rather than the simple prevention and control between geographically adjacent cities. The government departments should simultaneously consider cities’ statuses and their abilities of spatial spillover effects in the network when formulating relevant policies.

The remaining structure of this paper is organized as follows: The second section is the data and methods. The third section is the empirical results. The fourth section is the conclusion and policy implications.

## Data and Methods

### Data

**Figure 1:**
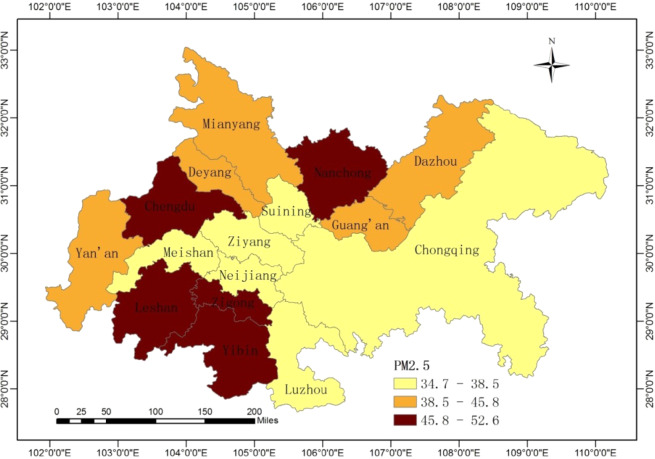
Relative geographical locations and yearly average PM_2.5_ values of the sixteen cities. The map was generated using ArcGIS 10.5. The source of the map is from national platform for common geospatial information services of China (http://www.mnr.gov.cn/sj/sjfw/).

The focus of the study area is Cheng-Yu urban agglomeration which is the fourth largest urban agglomeration and a comprehensive demonstration zone of new urbanization in China. It is composed of 16 cities including Chongqing, Chengdu, Zigong, Luzhou, Deyang, Mianyang, Suining, Neijiang, Leshan, Nanchong, Meishan, Yibin, Guang’an, Dazhou, Ya’an and Ziyang. Among them, Chongqing is a municipality directly under the central government, the other fifteen cities are located in Sichuan province, and Chengdu is the capital of Sichuan province. The relative geographical locations are shown in Fig. 1.

PM_2.5_ has already served as a guide for the governments to inform the public to take protection measures in a bid to avoid long-time exposure to haze pollution, and we use PM_2.5_ to illustrate urban haze pollution. The daily PM_2.5_ data for 16 cities from January 1 to December 31 in 2018 is obtained from China’s air quality online monitoring and analysis platform (https://www.aqistudy.cn/). It is a high-frequency data, involving a total of 365 observations. The descriptive statistics for PM_2.5_ of the sixteen cities (means, standard deviations (S.D), skewness, kurtosis) are presented in Table 1. Also, we apply the geovisualization technique to map the yearly average PM_2.5_ values of the sixteen cities. From Fig. 1, we find that cities in the north and southwest of Cheng-Yu urban agglomeration are heavily polluted. Obviously, Chengdu, Nanchong, Leshan, Zigong and Yibin face serious haze pollution.

**Table 1:** The descriptive statistics of PM_2.5_ (μg/m^3^).

city	Chongqing	Chengdu	Zigong	Luzhou	Deyang	Mianyang	Suining	Neijiang
Mean	38.15	49.44	52.60	37.75	41.62	45.34	34.85	37.25
S.D.	21.25	32.16	36.04	26.51	31.27	32.84	21.22	27.60
skewness	2.02	1.42	1.43	1.58	1.56	1.68	1.69	1.34
kurtosis	5.62	2.69	2.33	2.69	3.11	4.01	5.74	1.89
PP test	−6.37***	−6.61***	−5.23***	−5.95***	−6.28***	−5.77***	−6.62***	−5.22***
city	Leshan	Nanchong	Meishan	Yibin	Guang’an	Dazhou	Ya’an	Ziyang
Mean	45.92	46.83	38.48	50.68	40.32	45.84	39.56	34.73
S.D.	28.48	24.29	25.67	30.55	26.08	32.70	27.59	24.02
skewness	1.34	1.40	1.56	1.24	1.71	1.87	1.30	1.68
kurtosis	2.34	3.03	3.13	1.43	4.21	4.35	1.98	2.66
PP test	−6.99***	−6.59***	−6.38***	−6.83***	−6.39***	−6.31***	−6.53***	−4.82***

Notes:*Significance at 10% level; **Significance at 5% level; ***Significance at 1% level.

### Methods

#### Constructing haze pollution spatial association network

The Granger causality test^21^ provides an analytical tool for examining the spatial association of urban haze pollution from a time series perspective. This analytical tool has been widely used in economic, environmental pollution, climate change and other fields^5,14,22–24^. We use the Granger causality test to identify the spatial association of haze pollution between cities in Cheng-Yu urban agglomeration by constructing the following vector auto regression (VAR) model:

1 $${{\rm{x}}}_{t}={\alpha }_{1}+\mathop{\sum }\limits_{i=1}^{n}{\beta }_{i}{x}_{t-i}+\mathop{\sum }\limits_{j=1}^{n}{\gamma }_{j}{y}_{t-j}+{\mu }_{1,t}$$

2 $${y}_{t}={\alpha }_{2}+\mathop{\sum }\limits_{i=1}^{m}{\delta }_{i}{y}_{t-i}+\mathop{\sum }\limits_{j=1}^{m}{\theta }_{j}{x}_{t-j}+{\mu }_{2,t}$$

Among them, ${{\rm{x}}}_{t}$ and ${y}_{t}$ are the haze pollution (PM_2.5_) index sequences of city x and city y, respectively. α, β, γ, 𝛿, 𝜃 are parameters, m, n are lag orders determined according to AIC criterion, ${\mu }_{1,t}$, ${\mu }_{2,t}$ are random disturbance terms. By checking whether the parameters of the lag term x and y are all zero, the haze pollution relationship between city x and city y is determined (See Table 2.). This makes it possible to construct haze pollution spatial association network in Cheng-Yu urban agglomeration.

**Table 2:** Granger causality test.

Null hypothesis	Result	Null hypothesis	Result	Relationship
${\gamma }_{1}={\gamma }_{2}=\ldots ={\gamma }_{n}=0$	Rejected	${\theta }_{1}={\theta }_{2}=\ldots ={\theta }_{n}=0$	Accepted	x ← y
${\gamma }_{1}={\gamma }_{2}=\ldots ={\gamma }_{n}=0$	Accepted	${\theta }_{1}={\theta }_{2}=\ldots ={\theta }_{n}=0$	Rejected	x → y
${\gamma }_{1}={\gamma }_{2}=\ldots ={\gamma }_{n}=0$	Rejected	${\theta }_{1}={\theta }_{2}=\ldots ={\theta }_{n}=0$	Rejected	x ↔ y
${\gamma }_{1}={\gamma }_{2}=\ldots ={\gamma }_{n}=0$	Accepted	${\theta }_{1}={\theta }_{2}=\ldots ={\theta }_{n}=0$	Accepted	No

It should be noted that the Granger causality test is only applicable to the stationary sequence. Hence, we use the Phillips and Perron test (PP test) method to determine if PM_2.5_ of each city is a stationary time series.

#### Overall network structure

Network density is an indicator that reflects the tightness of the network. It is equal to the actual number of relationships divided by the maximum possible relationships in the network. If the number of cities in the urban agglomeration is N, the actual number of relationships is M, the formula for calculating the network density is as follows:

3 $${\rm{ND}}=\frac{M}{N(N-1)}$$

Network relevance is an indicator that reflects the robustness and vulnerability of the network structure. Let V be the number of pairs of cities that are unreachable in the network, then the network relevance can be calculated as follows:

4 $${\rm{NR}}=1-\frac{V}{N(N-1)/2}$$

Network efficiency is an indicator of the redundant relationship in the network. Let R be the number of extra lines and Max(R) the maximum number of possible extra lines, then the network efficiency can be calculated as follows:

5 $${\rm{NE}}=1-\frac{R}{{\rm{\max }}(R)}$$

Network grade is an indicator that reflects the dominance of members in the network. Let S be the number of pairs of cities symmetrically reachable in the network. Max(S) is the maximum number of pairs of cities that can be reached symmetrically in the network. The network grade can be calculated as follows:

6 $${\rm{NG}}=1-\frac{S}{{\rm{\max }}(S)}$$

#### Network centrality

Centrality is an indicator of the status and role of the city in haze pollution spatial association network. Freeman^25^ suggests that it can be characterized by point centrality, betweenness centrality, and closeness centrality.

Point centrality reflects the central position of the city in the network. It can be expressed as the relationships of city i directly associated with the target city. As haze pollution association network is a directed network, the degree of point centrality can be further divided into in-degree and out-degree to reflect the extent to which a city is affected by other cities and affect other cities.

Betweenness centrality reflects the ability of a city to control other cities in the network. Let ${b}_{jk}(i)$ be the ability of city i to control the relationship between city j and city k. Betweenness centrality can be calculated by the formula (7).

7 $$B{C}_{i}=\mathop{\sum }\limits_{j}^{N}\mathop{\sum }\limits_{k}^{N}{b}_{jk}(i),\,(j\ne k\ne i,\,j < k)$$

Closeness centrality reflects the extent to which a city is not controlled by other cities in the network. Let *d*_*ij*_ be the shortcut distance between city i and city j. Closeness centrality can be calculated by the formula (8).

8 $$C{C}_{i}^{-1}=\mathop{\sum }\limits_{j=i}^{N}{d}_{ij}$$

#### Block modelling

Block modelling is the main method of spatial clustering in social network analysis^26,27^. We apply block modelling to divide the role and status of each city in the haze pollution spatial association network into four types. The first type is net beneficial block. The members of this kind of block accept relationships from members of other block as well as from members of itself, while the relationships it receives from other blocks are obviously more than the relationships it sends to other blocks. The second type is net overflow block. This kind of block sends more relationships to other blocks than it receives from other blocks. The third type is bilateral overflow block. This kind of block both sends relationships to other blocks and accepts relationships from other blocks, however, there are relatively more relations between the members of itself. The fourth type is broker block. This kind of block both sends relationships to other blocks and accepts relationships from other blocks, however, it establishes relatively more relationships with other blocks.

#### QAP

Quadratic assignment procedure (QAP) analysis method has excellent characteristics that do not require independence between variables and frequently used for analyzing relational data. In this paper, we use QAP analysis method to reveal the impact of urbanization on spatial spillover of haze pollution. The basic principle of QAP regression analysis consists of three steps: First, the independent variable matrix and dependent variable matrix are transformed into long vectors, and the associaon coefficients are calculated along the long vectors. Secondly, a large number of association coefficients are calculated as the first step by randomly replacing the matrix of rows and corresponding columns thousands of times. Finally, the association coefficient between the independent variable matrix and dependent variable matrix is determined by the ratio of the association coefficients obtained, and the significance level is judged based on the distribution of association coefficients. According to the above theoretical analysis, the model constructed is as follows:

9 $${{\rm{P}}{\rm{M}}}_{2.5}=f(U)$$

Among them, PM_2.5_ is the spatial association matrix of haze pollution in Cheng-Yu urban agglomeration. U is the difference matrix of urbanization level. The level of urbanization is measured by the proportion of urban population in the total population. The data of urbanization is derived from Sichuan Statistical Yearbook 2019 and Chongqing Statistical Yearbook 2019.

## Empirical Results

### The results of overall network structure analysis

**Figure 2:**
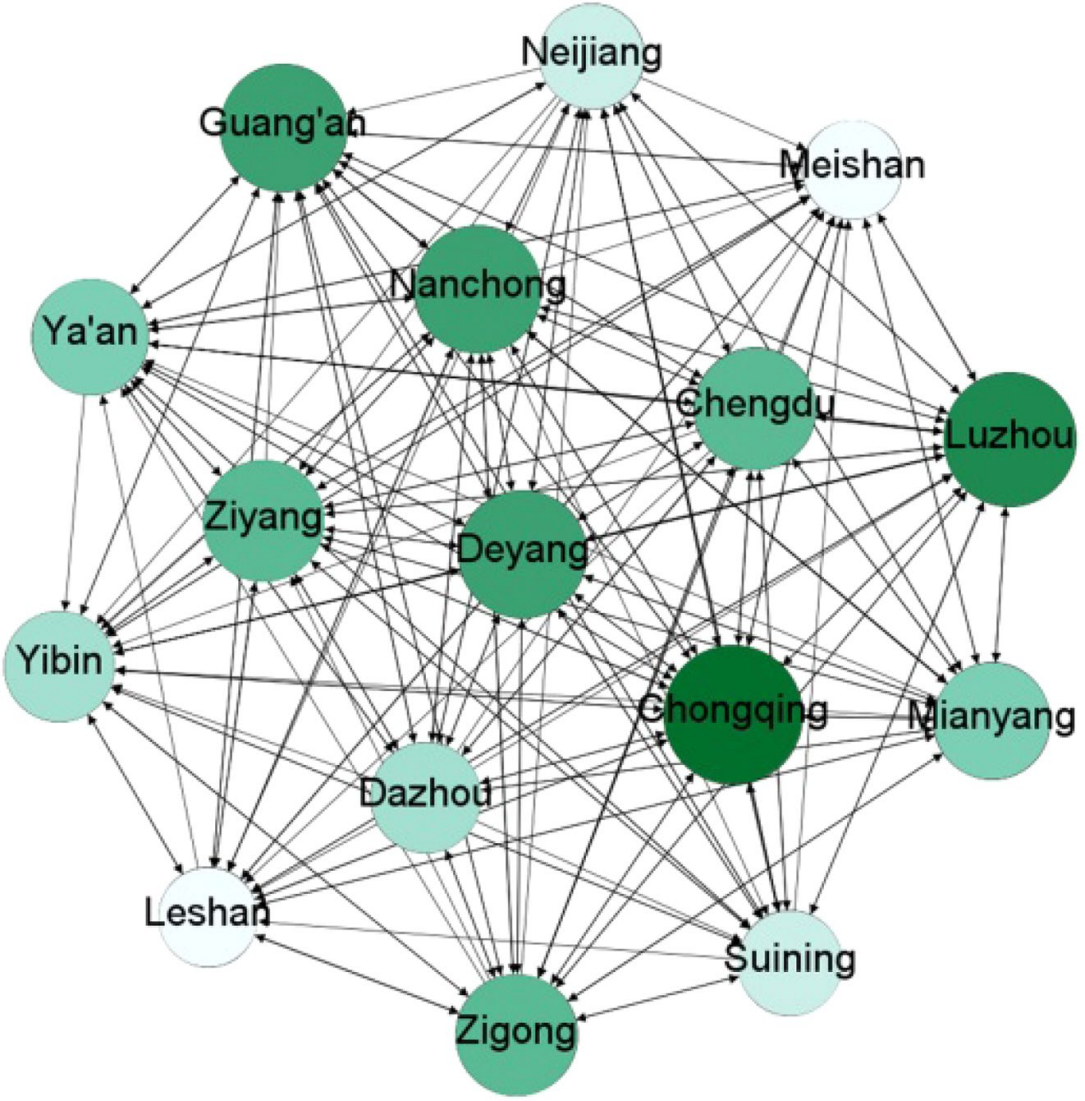
The topology diagram of haze pollution spatial association network. The topology diagram was generated using Gephi 0.9.2.

Before the granger causality test, we use the PP unit root test to conduct stability analysis on the daily PM_2.5_ series of each city. The results show that the PM_2.5_ series of all cities were stationary with the significance of 0.01(See Table 1). Next, we conduct a Granger causality test based on the VAR model between two cities in Cheng-Yu urban agglomeration. The AIC criterion is used to determine the lag order of the VAR model, and the VAR Granger causality Wald tests are used to judge the significance with 0.05 as the criterion. It turns out that there are 210 Granger causal relationships in 240 tests. Among them, there are 79 two-way Granger causality relationships and 26 one-way Granger causality relationships. According to the results of Granger causality test, the spatial network diagram of the Cheng-Yu urban agglomeration can be drawn, as shown in Fig. 2.

Furtherly, we calculate the overall network structure characteristics using formulae (3) to (6). The overall network density of the haze pollution spatial association network is 0.875, indicating that haze pollution is linked closely in Cheng-Yu urban agglomeration. The network relevance is 1, the network efficiency is 0.1, and the network grade is 0, indicating that the haze pollution spatial association network isn’t a strict hierarchical network and there is no isolated city. To sum up, Haze pollution is a complex spillover relationship in Cheng-Yu urban agglomeration and not a simple spillover relationship between adjacent cities. Haze pollution of each city is directly or indirectly affected by other cities in Cheng-Yu urban agglomeration.

### The results of network centrality analysis

Table 3 reports the results of network centrality of the haze pollution spatial association network. It turns out that Chongqing, Chengdu, Zigong, Deyang, Nanchong and Dazhou have the highest out-degree, all of which are 15, indicating that these cities send more relationships to other cities and they have stronger ability to influence other cities. In addition, the out-degree of Luzhou, Mianyang and Neijiang are also higher, indicating that these three cities are also more capable of exporting haze pollution. Chongqing, Luzhou, Guang’an, Yibin and Meishan have the highest in-degree, indicating that they accept more relationships and are more affected by other cities. In addition, the in-degree of Ziyang and Ya’an are also high, indicating that these two cities are also greatly affected by haze pollution from other cities.

**Table 3:** The results of network centrality.

City	Out-degree	In-degree	Betweenness	In-Closeness	Out-Closeness
Chongqing	15	15	3.63	100.00	100.00
Chengdu	15	12	1.25	83.33	100.00
Zigong	15	12	3.06	83.33	100.00
Luzhou	14	15	2.93	100.00	93.75
Deyang	15	13	1.91	88.24	100.00
Mianyang	14	12	2.00	83.33	93.75
Suining	12	12	1.04	83.33	83.33
Neijiang	14	10	0.69	75.00	93.75
Leshan	10	13	0.65	88.24	75.00
Nanchong	15	13	2.27	88.24	100.00
Meishan	8	15	0.76	100.00	68.18
Yibin	10	15	1.38	100.00	75.00
Guang’an	13	15	2.81	100.00	88.24
Dazhou	15	10	0.83	75.00	100.00
Ya’an	12	14	2.26	93.75	83.33
Ziyang	13	14	2.54	93.75	88.24

The city with the highest betweenness centrality is Chongqing, with a value of 3.63, indicating that Chongqing is the most important media city in the haze pollution spatial association network. In addition, the betweenness centrality of Zigong, Luzhou and Guang’an is 3.06, 2.93 and 2.81, respectively, indicating that these cities are also important media cities. Cities such as Leshan, Neijiang, Meishan and Dazhou have lower betweenness centrality, indicating that these cities are on the fringe of the network and are dominated by other cities.

The cities with the highest in-closeness are Chongqing, Luzhou, Guang’an, Yibin and Meishan, the cities with the highest out-closeness are Chongqing, Nanchong, Deyang, Zigong, Chengdu and Dazhou, indicating these cities are the core cities that are not easily controlled, and they have strong independence in the haze pollution spatial association network. Therefore, they have important demonstration value.

The network centrality analysis reveals that Chongqing, Chengdu, Guang’an, Luzhou, Deyang and Nanchong are located at the heart of the haze pollution spatial association network. Looking at these cities, it is easy to see that they are all heavy industrial cities. Although Chengdu and Chongqing, respectively, are the core cities with advanced policies, advanced economic leaders and advanced management in Cheng-Yu urban agglomeration, their statuses in the haze pollution spatial association network are not similar, Chongqing’s network status is higher, and has stronger influence in the network. Therefore, government departments should formulate policies adapted to their own conditions and geographic locations in the network.

### The results of block modelling analysis

Specifically, we use CONCOR analysis method to conduct the block modelling analysis. The maximum segmentation density is set to 2, the convergence criterion is set to 0.2, and the haze pollution spatial association network are divided into four blocks (See Table 4). Among them, the first block contains three cities, followed by Chongqing, Ziyang and Guang’an. The second block contains four cities, followed by Luzhou, Meishan, Yibin and Leshan. The third block contains six cities, followed by Chengdu, Suining, Dazhou, Deyang, Nanchong and Neijiang. The fourth block contains three cities, followed by Zigong, Ya’an and Mianyang.

**Table 4:** Spillover effect of spatial relationship block of haze pollution.

Block	The number of cities	Receive relationship		Send relationship		Expected internal relationship ratio	Actual internal relationship ratio	Block type
		Inside the block	Outside the block	Inside the block	Outside the block			
I	3	5	39	5	36	13%	12%	Bilateral overflow
II	4	11	47	11	31	20%	26%	Net beneficiary
III	6	28	42	28	58	33%	33%	Net overflow
IV	3	4	34	4	37	13%	10%	Broker

Notes: Expected internal relationship ratio = (number of cities within the block-1) / (number of cities in the network-1); Actual internal relationship ratio = number of internal relationships of blocks / total number of spillover relationships of blocks.

Table 4 reports the relationships between and within blocks. The results show that there are 48 relationships within the block and 162 relationships between blocks among the total 210 relationships. In the first block, there are 41 overflow relationships, among them, there are 5 relationships within the block and 36 relationships outside the block. This block receives 44 relationships from other blocks, among them, there are 5 relationships within the block and 39 relationships outside the block. The expected internal relationship is 13%, the actual internal relationship is 12%. The relationships receiving are obviously more than relationships sending. So, the first block is bilateral overflow block. In the second block, there are 42 overflow relationships, among them, there are 11 relationships within the block and 31 relationships outside the block. This block receives 58 relationships from other blocks, among them, there are 11 relationships within the block and 47 relationships outside the block. The expected internal relationship is 20%, the actual internal relationship is 26%. So, the second block is net beneficiary block. In the third block, there are 86 overflow relationships, among them, there are 28 relationships within the block and 58 relationships outside the block. This block receives 70 relationships from other blocks, among them, there are 28 relationships within the block and 40 relationships outside the block. The expected internal relationship is 33%, the actual internal relationship is 33%. So, the third block is net overflow block. In the fourth block, there are 41overflow relationships, among them, there are 4 relationships within the block and 37 relationships outside the block. This block receives 38 relationships from other blocks, among them, there are 4 relationships within the block and 34 relationships outside the block. The expected internal relationship is 13%, the actual internal relationship is 10%. So, the fourth block is broker block.

According to the block modelling analysis, the density matrix of the each block can be obtained. We take the overall network density of 0.875 as the 0-block and 1-block distinguishing point to establish the corresponding image matrix, so as to further analyze the spatial relationships between the haze pollution of various blocks. If the density of each block is greater than 0.875, the block has a concentrated trend and the value is 1, otherwise, the value is 0. From this, the image matrix is shown in Table 5. It can be found that the first block, the second block and the fourth block are the main overflow object for other blocks. The first block, the third block and the fourth block overflow more relationships to other blocks. It indicates that the cities of the first block, the third block and the fourth block are the main sources of haze pollution, and they should be the focus of haze pollution control, while the cities of the second block are the main victims of haze pollution.

**Table 5:** The density matrix and image matrix of haze pollution.

Block	Density matrix				Image matrix			
	I	II	III	IV	I	II	III	IV
I	0.833	1	0.889	0.889	0	1	1	1
II	1	0.917	0.375	0.833	1	1	0	0
III	1	1	0.933	0.889	1	1	1	1
IV	1	0.917	0.944	0.667	1	1	1	0

**Figure 3:**
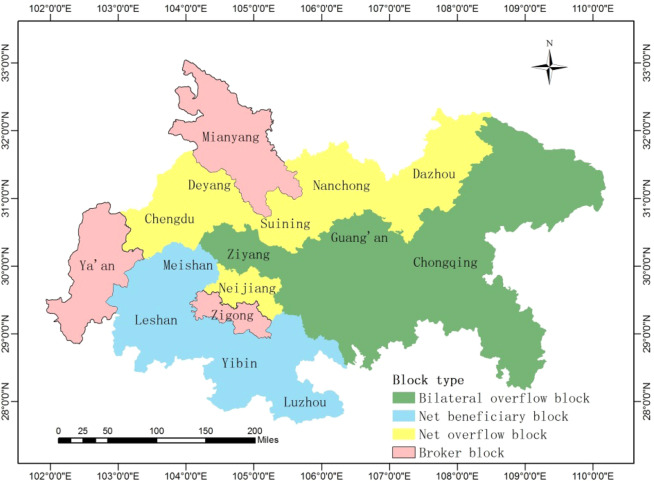
Spatial distribution of the four blocks. The map was generated using ArcGIS 10.5. The source of the map is from national platform for common geospatial information services of China (http://www.mnr.gov.cn/sj/sjfw/).

Furtherly, we map the spatial distribution of the four blocks in the haze pollution spatial association network and presented in Fig. 3. It can be found that cities belongs to net beneficiary block are mainly located in the southwest of Cheng-Yu urban agglomeration, they are the main receivers of haze pollution. Cities belongs to net overflow block are mainly located in the north of Cheng-Yu urban agglomeration, they are the main senders of haze pollution. Cities belongs to bilateral overflow block are mainly located in the east of Cheng-Yu urban agglomeration, while, cities belongs to broker block are relatively dispersed, and they are the main transmission channel of haze pollution.

### The results of QAP analysis

Table 6 reports the results of QAP regression analysis. It can be found that the unstandardized coefficient is 0.004, and the standardized regression coefficient is 0.105, passing the 0.1 significance level test. It indicates that the difference of urbanization has significant influence on the spatial spillover of haze pollution, the greater the difference of urbanization between cities, the stronger the spillover of haze pollution. In fact, industry is being concentrated in cities with the process of urbanization, which generates heavy pollution discharge when promoting the economic growth of cities. However, urbanization varies greatly between cities in Cheng-Yu urban agglomeration. Some cities with high urbanization benefited from economic development, but the resulting haze pollution was shared by all cities.

**Table 6:** The results of QAP regression analysis.

Variables	Untandardized coefficient	Standardized coefficient	P-value	As Large	As Small	Std Err
Urbanization	0.004	0.105	0.082	0.082	0.919	0.003
Intercept	0.840	0.000	0.000	0.000	0.000	0.000

Notes: The number of random permutations in QAP regression analysis is 2000 times.

**Figure 4:**
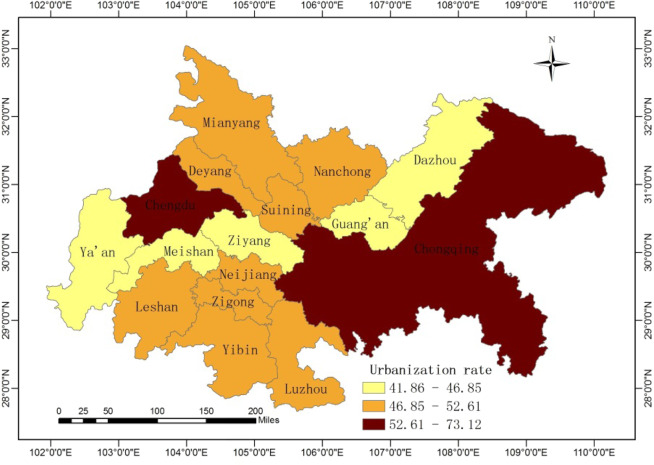
Spatial distribution of urbanization rate. The map was generated using ArcGIS 10.5. The source of the map is from national platform for common geospatial information services of China (http://www.mnr.gov.cn/sj/sjfw/).

Furtherly, we apply the geovisualization technique to map the level of urbanization in Cheng-Yu urban agglomeration. Analyzing Fig. 4, it displays that Chongqing and Chengdu have the highest urbanization rate, which was 65.5 percent and 73.12 percent in 2018, respectively. Meanwhile, they overflow more haze pollution relationships as described above. Although the urbanization difference between the north and the south of Cheng-Yu urban agglomeration is small, they establish more haze pollution relationships through media cities. In fact, cities with high urbanization level are also cities with severe haze pollution, so they should compensate the cities with low urbanization level suffering haze pollution.

## Conclusions and policy implications

In this study, our aim is to take a network perspective to examine the spatial spillover effects of haze pollution, analyze the network characteristics of haze pollution spatial association network, and reveal the impact of urbanization on spatial spillover of haze pollution in Cheng-Yu urban agglomeration. The main findings are as follows: First, we use the Granger causality test to construct the haze pollution spatial association network in Cheng-Yu urban agglomeration. It turns out that haze pollution is a complex multithreaded spatial association network beyond geographical proximity. Haze pollution of each city is directly or indirectly affected by other cities in Cheng-Yu urban agglomeration. Second, by the network centrality analysis, we find that Chongqing, Chengdu, Zigong, Deyang, Nanchong and Dazhou have stronger ability to influence other cities, and they are located at the heart of the haze pollution spatial association network. Although Chengdu and Chongqing are two core cities in Cheng-Yu urban agglomeration, their statuses in the haze pollution spatial association network are not similar, Chongqing’s network status is higher, and has stronger influence in the network. Third, the haze pollution spatial association network can be divided into four blocks: bilateral overflow block, net beneficiary block, net overflow block and broker block. Cities belongs to net beneficiary block are mainly located in the southwest of Cheng-Yu urban agglomeration, they are the main receivers of haze pollution. Cities belongs to net overflow block are mainly located in the north of Cheng-Yu urban agglomeration, they are the main senders of haze pollution. Cities belongs to bilateral overflow block are mainly located in the east of Cheng-Yu urban agglomeration, while, Cities belongs to broker block are relatively dispersed, and they are the main transmission channel of haze pollution. Fourth, the difference of urbanization has significant influence on the spatial spillover of haze pollution. Cities with higher level of urbanization overflow more relationships.

In response to the research conclusions, we propose some relevant policy recommendations for haze pollution control in Cheng-Yu urban agglomeration: First, government departments should earnestly trace the haze pollution source and transmission channel. By controlling the key cities in the haze pollution spatial association network, such as Chongqing, Chengdu, Zigong, Deyang, Nanchong and Dazhou, the haze pollution can be controlled effectively. Specially, government departments should simultaneously consider cities’ locations and their spatial spillover effects in the network when they formulate relevant policies. Second, the compensation mechanism between cities in Cheng-Yu urban agglomeration should be established. It is necessary to integrate the haze pollution control into coordinated development strategy of Cheng-Yu urban agglomerations. Different cities should bear different responsibilities in the joint control of haze pollution, and cities with high urbanization level should compensate the cities with low urbanization level suffering haze pollution by establishing stable fiscal environmental protection expenditures or haze pollution control fund. Third, take the comprehensive demonstration zone of new urbanization in China as an opportunity to upgrade the industrial structure, and encourage the highly-polluted secondary industry moving to the high-value-added service industry.
